# LTB4 Promotes Acute Lung Injury via Upregulating the PLC*ε*-1/TLR4/NF-*κ*B Pathway in One-Lung Ventilation

**DOI:** 10.1155/2022/1839341

**Published:** 2022-01-11

**Authors:** Jing Luo, Qingjie Ma, Heng Tang, Xi Zou, Xin Guo, Yuzhen Hu, Kejiang Zhou, Rui Liu

**Affiliations:** ^1^Department of Pain Management, The First People's Hospital of Yunnan Province, China; ^2^Department of Anesthesiology, The First People's Hospital of Yunnan Province, China

## Abstract

**Background:**

Mechanical ventilation (MV) can provoke acute lung injury (ALI) by increasing inflammation activation and disrupting the barrier in lung tissues even causing death. However, the inflammation-related molecules and pathways in MV-induced ALI remain largely unknown. Hence, the purposes of this study are to examine the role and mechanism of a novel inflammation-related molecule, leukotriene B4 (LTB4), in ALI.

**Methods:**

The functions of LTB4 in one-lung ventilation (OLV) model were detected by the loss-of-function experiments. H&E staining was used to examine the pathologic changes of lung tissues. Functionally, PLC*ε*-1 knockdown and Toll-like receptor 4 (TLR4)/NF-*κ*B pathway inhibitor were used to detect the regulatory effects of LTB4 on the phospholipase C*ε* (PLC*ε*-1)/TLR4/nuclear factor-kappa B (NF-*κ*B) pathway. The levels of genes and proteins were determined by RT-qPCR and western blotting assay. The levels of inflammation cytokines and chemokines were measured by ELISA.

**Results:**

Here, we found LTA4H, leukotriene B (4) receptor 1 (BLT1), LTB4, and PLC*ε*-1 upregulated in OLV rats and associated with inflammatory activation and lung permeability changes of lung tissues. Inhibition of LTB4 alleviated the OLV-induced ALI by inhibiting inflammatory activation and lung permeability changes of lung tissues. For mechanism analyses, LTB4 promoted OLV-induced ALI by activating the PLC*ε*-1/TLR4/NF-*κ*B pathway.

**Conclusion:**

LTB4 induced ALI in OLV rats by activating the PLC*ε*-1/TLR4/NF-*κ*B pathway. Our findings might supply a new potential therapeutic for OLV-induced ALI.

## 1. Introduction

Acute lung injury (ALI) is a common acute respiratory distress syndrome (ARDS) mostly caused by supporting mechanical ventilation (MV) [1, 2]. ALI is the dominant cause of death induced by thoracic surgery [3]. The pathological changes include mechanical injury in the endotracheal wall and promote inflammatory activation in lung tissues. The mechanical injury of MV includes edema-flooded airways and unstable alveoli, which results in enhancing the permeability of pulmonary microvascular endothelial cells (PMVECs) and lung epithelial cells (LECs) [4, 5]. Usually, the damage of PMVECs and LECs has been induced by immune cell infiltrating. The inflammatory activity is mediated by the heavy increasing of neutrophils in lung tissues; the infiltrated neutrophils produce proinflammatory cytokines including interleukin-1*β* (IL-1*β*), IL-6, IL-8, and tumor necrosis factor-*α* (TNF-*α*) and attract chemokines such as C-X-C motif ligands (CXCLs) and C-C motif ligands (CCLs) [6]. On the one hand, neutrophils promote airway wall remodeling by excessing expression of matrix metalloproteinase-9 (MMP-9) and neutrophil elastase [7]. On the other hand, neutrophils recruit immune cells such as leukocytes and promote the adaptive immune responses [8]. Taken together, the above researches reveal that neutrophil plays a critical role in ALI through promoting proinflammatory cytokine production, chemokine attraction, and immune cell recruitment in lung tissues to affect the permeability of lung.

Leukotriene B4 (LTB4) is mostly secreted by neutrophils and enhances the recruitment of neutrophils during inflammatory activity [9]. The procedures of LTB4 generation include 5-lioxygenase (5-LO), which migrates to the nucleus and interacts with 5-LO activating protein (FLAP) to promote arachidonic acid (AA) generation by leukotriene A4 (LTA4), and then, LTB4 is generated from LTA4 hydrolyzed by LTA4 hydrolase (LTA4H) [10]. Previous studies indicate that LTB4 accelerates neutrophil trend and swims to the injury tissue sites [11, 12]. It also indicates that increasing LTB4 promotes acute pancreatitis-associated lung injury by inducing neutrophil transendothelial migration [13]. Previous studies indicate LTB4 acts a key role in ALI by interfering neutrophil behavior. However, the underlying mechanism of LTB4 in ALI is largely unknown.

Not only LTB4 but also phospholipase C*ε* (PLC*ε*-1) acts a key role in neutrophil-mediated inflammation in ALI. Commonly, PLC*ε*-1 is a member of the phospholipase (PLC) family and plays as a second messenger by generating inositol 1,4,5-trisphosphate (IP3) and diacylglycerol (DAG) [14]. The Ca^2+^ generation and protein kinase C (PKC) activation were further promoted by IP3 and DAG, respectively [15]. PLC*ε*-1 mainly includes Ras association (RA) domain and guanine nucleotide exchange factor (EGF) domain, which is regulated by Ras family members and regulates the activity of small GTPase and Rap [16, 17]. It has been reported that PLC*ε* promotes cell proliferation of renal cell carcinoma (RCC) by activating the nuclear factor-kappa B (NF-*κ*B) pathway [18]. In addition, PLC*ε* enhances tumorigenesis and process by inducing proinflammatory cytokine expression in colon epithelial cells via activating the NK-*κ*B pathway [19]. The NF-*κ*B pathway is a classic proinflammatory signaling pathway involved in the upregulation of cytokines, chemokines, and adhesion molecules during inflammation [20]. The system of NF-*κ*B is defined by the interaction between NF-*κ*B dimers, I*κ*B regulators, and IKK complexes. The main compounds of the NF-*κ*B family include RelA (p65), RelB, cRel, NF-*κ*B1 (p105/p50), and NF-*κ*B2 (p100/p52), which are associated with homo- or heterodimerization and bind with DNA, respectively [21]. Increasing evidences have demonstrated that lipopolysaccharide (LPS) induced ALI by activating the NF-*κ*B pathway [22–24]. Despite larger researches indicating that the NF-*κ*B pathway may function a vital role in ALI, the regulatory mechanism of the NF-*κ*B pathway in ALI has not been clear.

Therefore, in this study, we examined the role of LTB4 and investigated the interaction between LTB4, PLC*ε*-1, and NF-*κ*B pathway in ALI. And we found that inhibition of LTB4 significantly reduced ALI by repressing the PLC*ε*-1/TLR4/NF-*κ*B pathway in one-lung ventilation (OLV) rat model.

## 2. Methods

### 2.1. Chemicals and Reagents

LTA4H inhibitor bestatin (HY-B0134) and TLR4/NF-*κ*B pathway inhibitor okanin (HY-N6673) were purchased from MedChemExpress (USA). Primary antibodies and secondary antibody goat anti-rabbit H&L were proved by Abcam (CA, USA).

### 2.2. Construction of OLV Rat Model

A total of thirty-six six-week-old male Sprague-Dawley (SD) rats with 200-220 g bodyweight were randomly divided into six groups (*n* = 6/group). One-lung ventilation (OLV) model was constructed according to the description of previous study [25]. Briefly, rats were anesthetized via intraperitoneally injecting 40 mg/kg pentobarbital. The trachea was exposed and placed with an endotracheal tube with inner diameter 3 mm, and then, the rats were connected to a ventilator (Harvard Apparatus, USA) with 3 h right lung mechanical ventilation. The parameters of the ventilator were set as follows: tidal volume (VT) = 10 mL/kg, inspiration expiration (I : E) ratio = 1 : 1, inspired oxygen concentration (FiO_2_), 100%. Respiratory rate (RR) = 40 times/min. 0.5% sodium pentobarbital with a rate of 4 mL/kg/h was used for maintenance anesthesia. Rats were randomly divided into six groups, including control group, OLV group, OLV+bestatin group, OLV+sh-NC group, OLV+sh-PLC*ε*-1 group, and OLV+okanin group. The rats of the bestatin group were treated following the described procedure before [26]. OLV+bestatin group rats intraperitoneally accepted 30 mg/kg bestatin at 15 min after surgery. OLV+okanin group rats accepted 50 mg/kg okanin at 15 min after surgery. OLV+sh-PLC*ε*-1 and OLV+sh-NC groups rats accepted 10 *μ*L shRNA (0.2 mg/mL) at 15 min after surgery. And the short hairpin RNA (shRNA) of PLC*ε*-1 and its negative shRNA were designed and inserted into the pAd/BLOCK-iT™-DEST vector (Thermo Fisher, CA, USA) according to the manuscript instruction. All animal experiments in this study were approved by the Animal Care and Use Committee and the Ethics committee of the First People's Hospital of Yunnan Province (2017YY067).

### 2.3. Quantitative RT-PCR

Total RNA was extracted from the superior lobe of left lung tissues using a Trizol reagent (TaKaRa, China) according to the manufacturer's protocol. And the reverse DNA was amplified using a PrimeScript™ 1st Strand cDNA Synthesis Kit (TaKaRa, China) following the manual of the manufacturer. And the quantitative PCR was performed using a TB Green® Fast qPCR Mix (TaKaRa, China) according to the manufacturer's instructions. The primers in this study are shown in Table 1. The relative expression of transcription was assessed using 2^-*ΔΔ*Ct^ methods. GAPDH was used as an internal control for gene expression.

### 2.4. Hematoxylin-Eosin (H&E) Staining

The part of the upper lobe of the right lung tissues was collected and fixed with 4% paraformaldehyde (PFA) and embedded with paraffin, and then, the tissues were sliced into 4 *μ*m and transferred onto glass slides. Furthermore, the slides were stained using an H&E staining kit (Beyotime, China) according to the manufacturer's protocol. And lung injury was calculated by injury scores based on the presence of atelectasis, hyaline membrane formation, neutrophil infiltration or aggregation into airspace, alveolar congestion, and hemorrhage: 0, minimal or no damage; 1, mild damage; 2, 25%-50% moderate damage; 3, 50%-75% severe damage; and 4, more than 75% maximal damage.

### 2.5. Lung Wet/Dry Ratio

The left lower lobes of lung tissues were harvested, and the weight was immediately measured. And then, the lung tissues were dehydrated in an incubator with 80°C for 72 h and the weight of the dry lung tissues was calculated. The wet/dry (W/D) ratio was evaluated according to [weight (wet) − weight (dry)]/weight (dry).

### 2.6. Enzyme-Linked Immunosorbent Assay (ELISA)

The levels of GM-CSF, IL-1*β*, IL-8, KC, MCP-1, MIP-2, TNF-*α*, and LTB4 in bronchoalveolar lavage fluid (BALF) or cell supernatants were measured by ELISA. Briefly, the part of the upper lobe of the right lung tissues was collected and homogenized with solution. The homogenates or cell-cultured media were centrifuged, and the levels of supernatants, homogenates, or cell-cultured media were measured using the ELISA kit (Solarbio, China) following the manual of manufacturers. The sensitivity of GM-CSF (DY528, R&D, US, USA), IL-1*β* (RLB00, R&D, US, USA), IL-8 (antibodies, Aachen, Germany), KC (RCN100, R&D, US, USA), MCP-1 (DY3144-05, R&D, US, USA), MIP-2 (RCN300, R&D, US, USA), TNF-*α* (DY510, R&D, US, USA), and LTB4 (520111, Cayman Chemicals, MI, USA) was 7.8 pg/mL, 5 pg/mL, 31.25 pg/mL, 1.3 pg/mL, 7.8 pg/mL, 2.7 pg/mL, 62.5 pg/mL, and 3.9 pg/mL.

### 2.7. Western Blotting Assay

Protein was isolated from the right lower lobe of lung tissues using a RIPA buffer (Beyotime, China) obeying the protocol of the manufacturer. Protein was then separated on 10% SDS-PAGE, transferred onto PVDF members (Millipore, USA), blocked with 5% nonfat milk, and probed with primary antibodies, such as PLC*ε*-1 (ab109501, Abcam, USA), TLR4 (#14358, Cell Signaling Technology, USA), RelA (p65, #3039, Cell Signaling Technology, USA), caspase 3 (#9662, Cell Signaling Technology, USA), Bcl-2 (ab194583, Abcam, USA), Bax (ab32503, Abcam, USA), MLCK (ab76092, Abcam, USA), VE-cadherin (ab231227, Abcam, USA), occludin (ab216327, Abcam, USA), ZO-1 (ab191143, Abcam, USA), and GAPDH (ab8245, Abcam, USA) at 4°C overnight, respectively. Subsequently, the members were incubated with the secondary antibody goat anti-rabbit IgG HRP (ab6721, Abcam, USA) at room temperature for 1 h. Finally, the bands were visualized using an enhanced chemiluminescent agent (Bio-Rad, USA). The levels of protein were normalized using GAPDH.

### 2.8. Identification of Inflammatory Cells in BALF

The number of PMNs and WBCs in BALF was assessed using a Diff-Quik staining assay according to the described procedure before [27]. Briefly, the BALF was centrifuged at 3000 rpm for 15 min and resuspended with 100 *μ*L cold PBS. And the number of cells was counted using a hemocytometer. And the smears were made using cytocentrifugation (Thermo Fisher Scientific, USA) and visualized using Diff-Quik staining kit (Sysmex Co., Japan). At least 200 cells on each slide were calculated under a microscope according to the stained phenotype of macrophages, neutrophils, lymphocytes, and eosinophils.

### 2.9. Statistical Analysis

All values in this study were represented as mean ± standard deviation (SD). And the statistical analysis was performed using GraphPad Prism 9.0. Differences between two groups or multiple groups were calculated using Student's *t*-test or one-way ANOVA. *P* value < 0.05 was identified as significant difference.

## 3. Results

### 3.1. OLV Induces Inflammatory Activation and Lung Permeability Changes

**Figure 1:**
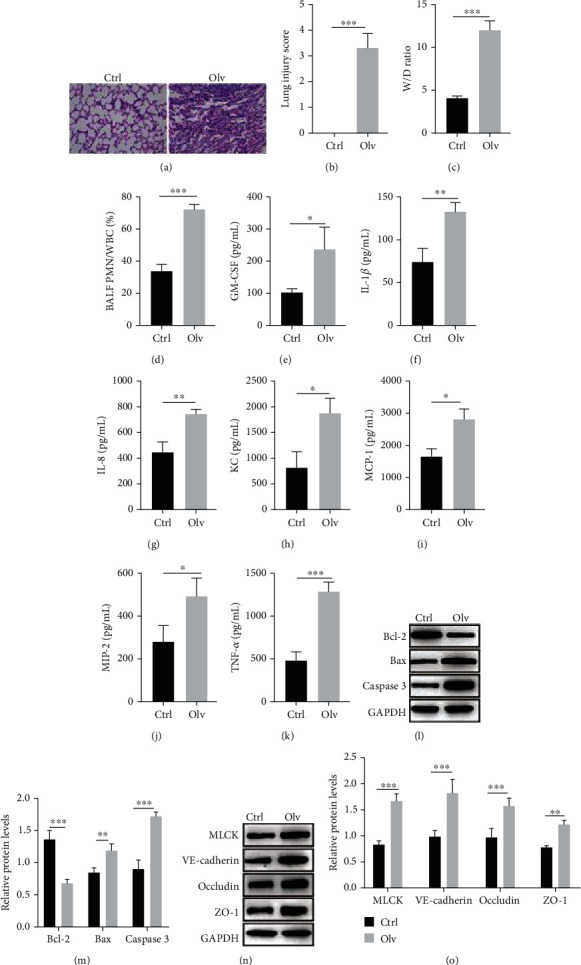
OLV induces inflammatory activation and lung permeability changes. (a, b) H&E staining detected the pathology of lung tissues in OLV and control rats. (c) The lung W/D ratio between OLV and control rats was calculated according to the formula [weight (wet) − weight (dry)]/weight (dry). (d) The PMN/WBC ratio of BALF in OLV and control rats was calculated. (e–k) ELISA performed to examine the levels of inflammation mediators (GM-CSF, IL-1*β*, IL-8, KC, MCP-1, MIP-2, and TNF-*α*) between the OLV group and control rats. (l–o) Western blot detected the protein levels of Bcl-2, Bax, caspase 3, MLCK, VE-cadherin, occludin, and ZO-1 in OLV and control rats. ^∗^*P* < 0.05, ^∗∗^*P* < 0.01, and ^∗∗∗^*P* < 0.001.

The OLV rat model was constructed, and H&E staining results revealed severe damage of lung tissues in OLV rats with obvious neutrophil infiltration into airspace, alveolar congestion, and hemorrhage compared with control rats (Figures 1(a) and 1(b)). The W/D ratio and the PMN/WBC ratio in BALF increased in OLV rats than control rats (Figures 1(c) and 1(d)), which indicated inflammation in OLV rats. Besides, the ELISA analyses showed increasing levels of cytokines and chemokines, including GM-CSF, IL-1*β*, IL-8, KC, MCP-1, MIP-2, and TNF-*α* in BALF of OLV rats than control rats (Figures 1(e)–1(k)). Western blot results also showed that the expression of Bax, caspase 3, MLCK, VE-cadherin, occludin, and ZO-1 was upregulated, and the expression of Bcl-2 was reduced in lung tissues from OLV rats (Figures 1(l)–1(o)). Our findings revealed that OLV induced acute lung injury by promoting inflammatory activation and pulmonary microvascular endothelial damage.

### 3.2. Upregulation of LTB4 in OLV-Induced ALI

**Figure 2:**
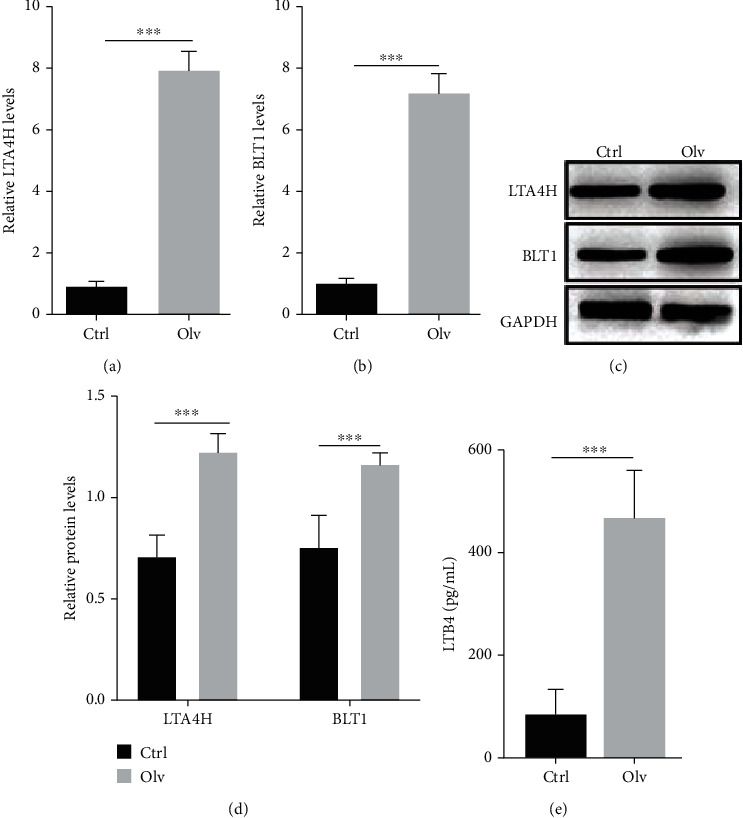
Upregulation of LTB4 in OLV-induced ALI. (a, b) qRT-PCR validated expression of LTA4H and BLT1 in OLV and control rats. (c, d) Western blot was performed to examine expression of LTA4H and BLT1 in OLV and control rats. (e) ELISA examined LTB4 level in OLV and control rats. ^∗∗^*P* < 0.01 and ^∗∗∗^*P* < 0.001.

LTB4 is generated from LTA4H and mediated by BLT1 to involve inflammation [28]. Here, we examined the expression of LTA4H, BLT1, and LTB4 in OLV rats. The qPCR and western blot results showed the mRNA and protein levels of LTA4H and BLT1 increased in lung tissues of OLV rats (Figures 2(a)–2(d)). ELISA results also indicated that increasing LTB4 level was observed in OLV rats (Figure 2(e)). These data indicated that LTB4 levels increased in OLV-induced ALI.

### 3.3. Inhibiting LTB4 Alleviates the OLV-Induced ALI

**Figure 3:**
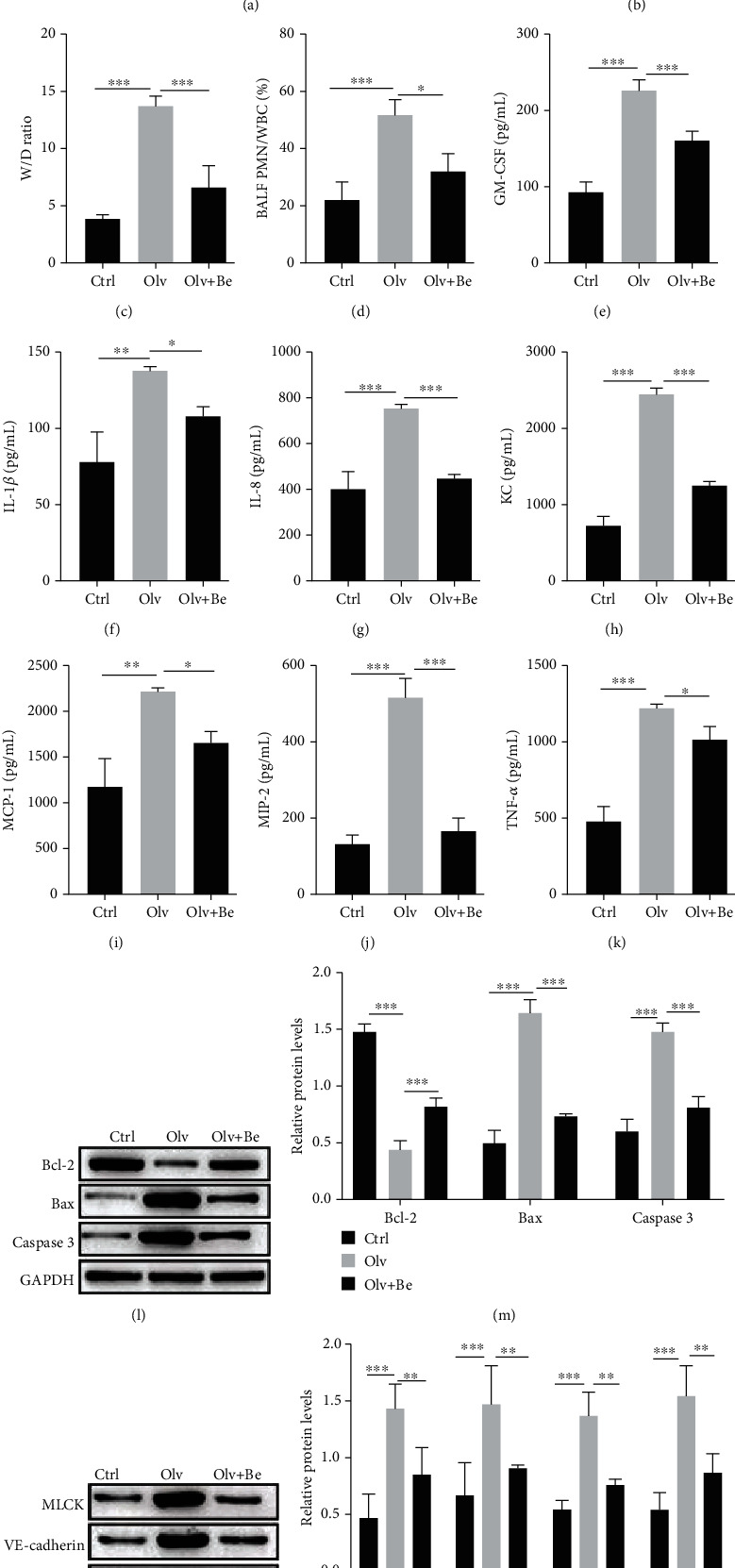
Inhibiting LTB4 alleviates the OLV-induced ALI. (a, b) H&E staining examined the pathology of lung tissues in control, OLV, and bestatin-treated rats. (c) The lung W/D ratio of lung tissues in control, OLV, and bestatin-treated rats was calculated. (d) The PMN/WBC ratio of BALF in control, OLV, and bestatin-treated rats was detected. (e–k) ELISA performed to examine the levels of inflammation mediators (GM-CSF, IL-1*β*, IL-8, KC, MCP-1, MIP-2, and TNF-*α*) in control, OLV, and bestatin-treated rats. (l–o) Western blot detected the protein levels of Bcl-2, Bax, caspase 3, MLCK, VE-cadherin, occludin, and ZO-1 in control, OLV, and bestatin-treated rats. ^∗^*P* < 0.05, ^∗∗^*P* < 0.01, and ^∗∗∗^*P* < 0.001.

We further examined the role of LTB4 in OLV-induced rats by using bestatin, which is a potent inhibitor of LTA4H. As shown in Figures 3(a) and 3(b), H&E staining results indicated severe damage of lung tissue in OLV rats, whereas bestatin alleviated the damage of lung tissue compared with OLV rats. Bestatin also reduced W/D ratio and the PMN/WBC ratio in BALF of OLV rats (Figures 3(c) and 3(d)). The increasing levels of cytokines and chemokines in OLV rats were reduced by bestatin (Figures 3(e)–3(k)). We also observed bestatin reduced the protein levels of Bax, caspase 3, MLCK, VE-cadherin, occludin, and ZO-1, but increased the protein levels of Bcl-2 in OLV rats (Figures 3(l)–3(o)). These data suggested that inhibition of LTB4 by bestatin alleviated the ALI in OLV rats.

### 3.4. Inhibiting LTB4 Reduces PLC*ε*-1 Expression

**Figure 4:**
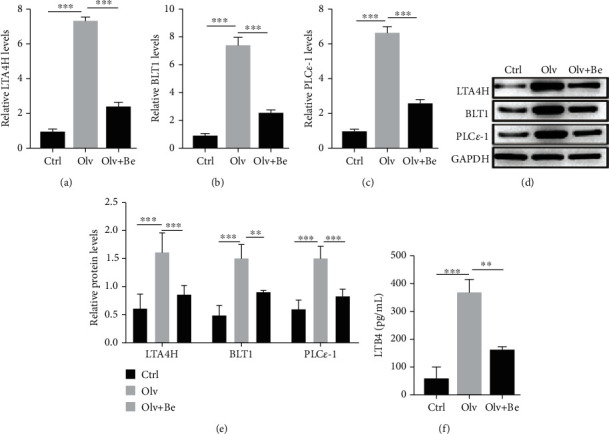
Inhibiting LTB4 reduces PLC*ε*-1 expression. (a–c) qRT-PCR validated expression of LTA4H, BLT1, and PLC*ε*-1 in control, OLV, and bestatin-treated rats. (d, e) Western blot was performed to examine expression of LTA4H, BLT1, and PLC*ε*-1 in control, OLV, and bestatin-treated rats. (f) ELISA examined LTB4 level in control, OLV, and bestatin-treated rats. ^∗∗^*P* < 0.01 and ^∗∗∗^*P* < 0.001.

We deeply investigated the potential mechanism of LTB4 in OLV rats. qPCR and western blot results indicated that bestatin not only reduced the mRNA and protein levels of LTA4H and BLT1 (Figures 4(a)–4(e)) and the levels of LTB4 (Figure 4(f)) but also reduced the mRNA and protein levels of PLC*ε*-1 in OLV rats. Our findings suggested that inhibition of LTB4 by bestatin reduced PLC*ε*-1 expression in OLV rats.

### 3.5. LTB4 Promotes OLV-Induced ALI by Activating the PLC*ε*-1/TLR4/NF-*κ*B Signaling Pathway

**Figure 5:**
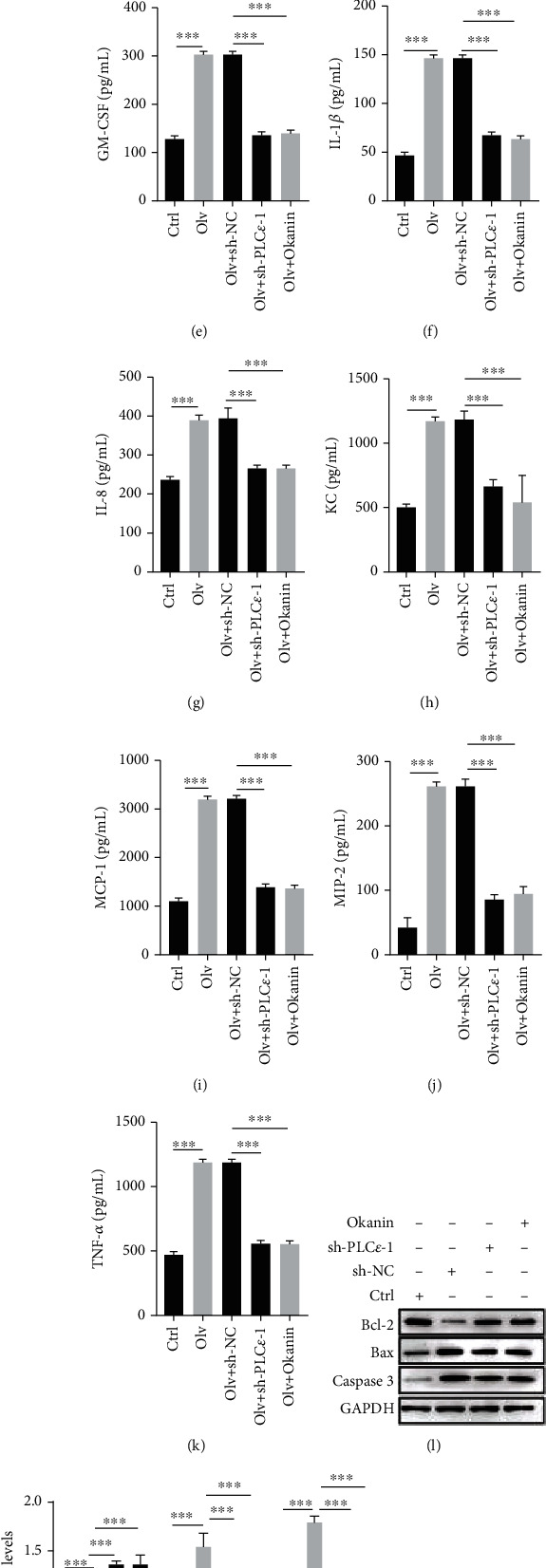
Inhibition of the PLC*ε*-1/TLR4/NF-KB signaling pathway reduced OLV-induced ALI. (a, b) H&E staining examined the pathology of lung tissues in control rats, OLV rats, PLC*ε*-1 knockdown rats, and okanin-treated rats. (c) The lung W/D ratio of lung tissues in control rats, OLV rats, PLC*ε*-1 knockdown rats, and okanin-treated rats was calculated. (d) The PMN/WBC ratio of BALF in control rats, OLV rats, PLC*ε*-1 knockdown rats, and okanin-treated rats was detected. (e–k) ELISA performed to examine the levels of inflammation mediators (GM-CSF, IL-1*β*, IL-8, KC, MCP-1, MIP-2, and TNF-*α*) in control rats, OLV rats, PLC*ε*-1 knockdown rats, and okanin-treated rats. (l–o) Western blot detected the protein levels of Bcl-2, Bax, caspase 3, MLCK, VE-cadherin, occludin, and ZO-1 in control rats, OLV rats, PLC*ε*-1 knockdown rats, and okanin-treated rats. ^∗^*P* < 0.05, ^∗∗^*P* < 0.01, and ^∗∗∗^*P* < 0.001.

**Figure 6:**
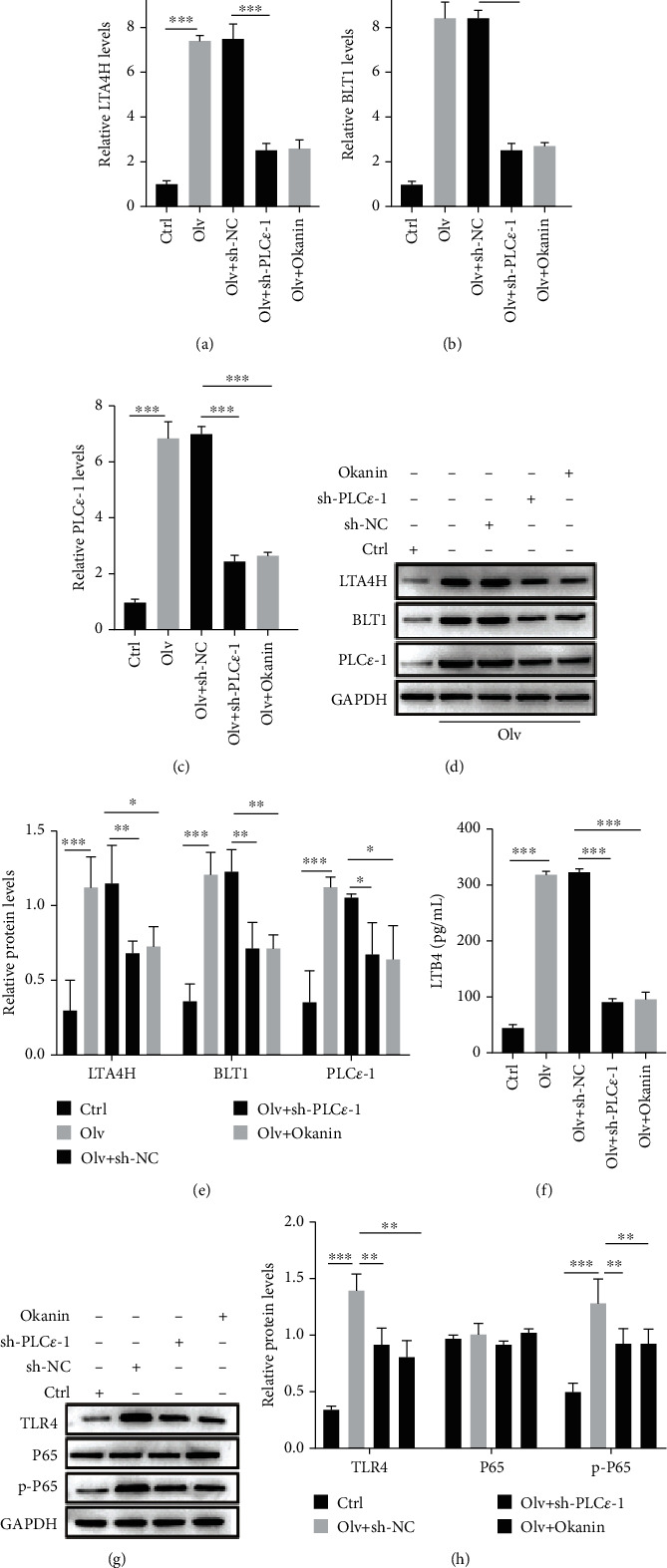
LTB4 promotes OLV-induced ALI by activating the PLC*ε*-1/TLR4/NF-KB signaling pathway. (a–c) qRT-PCR validated expression of LTA4H, BLT1, and PLC*ε*-1 in control rats, OLV rats, PLC*ε*-1 knockdown rats, and okanin-treated rats. (d, e) Western blot examined expression of LTA4H, BLT1, and PLC*ε*-1 in control rats, OLV rats, PLC*ε*-1 knockdown rats, and okanin-treated rats. (f) ELISA examined LTB4 level in control rats, OLV rats, PLC*ε*-1 knockdown rats, and okanin-treated rats. (g, h) Western blot detected expression of TLR4, P65, and phosphorylation of P65 in control rats, OLV rats, PLC*ε*-1 knockdown rats, and okanin-treated rats. ^∗^*P* < 0.05, ^∗∗^*P* < 0.01, and ^∗∗∗^*P* < 0.001.

The TLR4/NF-*κ*B signaling pathway is an important inflammatory pathway in lung injury [25, 29]. We detected whether LTB4 induced ALI in OLV rats by activating the PLC*ε*-1/TLR4/NF-*κ*B signaling pathway. Here, we found PLC*ε*-1 knockdown or TLR4/NF-*κ*B pathway inhibitor okanin significantly alleviated the damage of lung tissues in OLV rats (Figures 5(a) and 5(b)). PLC*ε*-1 knockdown or okanin also reduced the W/D ratio and the PMN/WBC ratio in BALF and the levels of cytokines and chemokines in OLV rats (Figures 5(c)–5(k)). PLC*ε*-1 knockdown or okanin decreased the protein levels of Bax, caspase 3, MLCK, VE-cadherin, occludin, and ZO-1, but upregulated the protein levels of Bcl-2 in OLV rats (Figures 5(l)–5(o)). Furthermore, we also found PLC*ε*-1 knockdown or okanin inhibited the mRNA and protein levels of LTA4H, BLT1, and PLC*ε*-1 (Figures 6(a)–6(e)) and reduced the levels of LTB4 in OLV rats (Figure 6(f)). Additionally, the TLR4 level and phosphorylation of p65 remained inhibited by PLC*ε*-1 knockdown or okanin (Figures 6(g) and 6(h)). These data indicated that inactivating the PLC*ε*-1/TLR4/NF-*κ*B signaling pathway relieved the ALI in OLV rats.

## 4. Discussion

Mechanical ventilation (MV) is a common strategy for emergency treatment in clinics and causes ALI or ARDS. Mostly, ALI has characteristics with pulmonary hypoxaemia, edema, and inflammation [30]. The pathophysiology of ALI contains neutrophil recruitment, release of proinflammatory cytokines and chemokines, and permeability change of PMVECs and LECs [31]. Inflammatory activity seriously promotes the process of ALI. Moreover, LTB4 has been found to be involved in the upregulation of ALI and is associated with neutrophil migration to lung tissues [32]. However, there is little known about the underlying mechanism of LTB4 regulating inflammatory activity in ALI. Therefore, we deeply explored the regulatory effect of LTB4 and its mechanism in ALI.

In the present study, we found LTB4 was upregulated in OLV rat model and promoted neutrophil and leukocyte recruitment to lung tissue, release of proinflammatory cytokines (TNF-*α*, IL-1*β*, and IL-8) and chemokines (CXCL1, MCP-1, and MIP-2) and GM-CSF, and induction of lung permeability via activating PLC*ε*-1/TLR4/NF-*κ*B cascaded signaling pathway.

LTB4 has been reported to function as an inflammatory promoter in neutrophil recruitment to lung sites, also affecting neutrophil migration and apoptosis in ALI [33]. Upregulation of LTB4 enhances cytokine and chemokine activation and recruitment of neutrophil to focus of inflammation [34]. In addition, LTB4 induces immune cell infiltration, such as monocytes (macrophages) and eosinophils (NK cells, T cells, T8 cells, and T4 cells) that generate the production of IL-1, IL-6, IL-8, and TNF-*α* to prolong inflammation in lung tissues [35]. Our results confirmed that the previous researches reveal LTB4 accelerates inflammatory activity through recruiting neutrophils and promoting cytokine and chemokine release. It is known to us that PLC-*ε* acts a proinflammatory role in ALI by inducing PMVEC permeability change and barrier disruption by activating proinflammatory cytokines and chemokines of neutrophils [14]. Previous studies have demonstrated that LTB4 recruits and activates of neutrophils, monocytes, and eosinophils by activating PLC [36, 37]. However, the interaction between LTB4 and PLC-*ε* in ALI has been unknown. In this study, we firstly proved the correlation between LTB4 and PLC-*ε* in ALI by activating neutrophils.

CXCL8 (IL-8) activation acts a vital role in the accumulation of neutrophils in the PMVECs of the lung tissue [38]. Furthermore, CXCL8 was increased in leucocyte in acute and chronic inflammatory diseases [39]. It also reports that CXCL8 generated from pulmonary epithelial cells functions an important role in the inflammation [40]. In this study, we found an accumulation of CXCL8 in BALF. Previous research has indicated that CXCL8 is involved in acute inflammatory disease by regulating the NF-*κ*B pathway [41]. Besides, CXCL8 induces lung inflammation in asthma by activating the NF-*κ*B pathway [42]. In our study, we proved that release of CXCL8 enhanced NF-*κ*B pathway activity. TLR4 is a receptor for LPS, which acts a vital role in innate and adaptive immune responses [43]. It also plays a key role in cellular stress and immune response in ALI by activating the NF-*κ*B pathway and releasing the proinflammatory factors IL-1*β*, IL-6, and TNF-*α* to further promote lung injury [23]. It has been demonstrated that inhibition of TLR4/NF-*κ*B pathway activation attenuates the inflammation in ALI [24].

## 5. Conclusion

Taken together, in this study, we explored a defined regulatory pathway which is the LTB4/PLC*ε*/CXCL8/TLR4/NF-*κ*B signaling pathway in ALI. Our findings might provide a novel potential therapeutic target for ALI and supply the novel insight to investigate neutrophil-mediated inflammatory diseases.

## Figures and Tables

**Table 1:** The sequence primers in this study.

Gene symbol	Sequence (5′-3′)
LTA4H	Forwards, 5′-CAGAGCCATGCCCGAGATAG-3′
	Reverse, 5′-GCAAATGTGGCGTTCCAAGT-3′
BLT1	Forwards, 5′-GTACCGCACAGTAACACCCA-3′
	Reverse, 5′-AGTCATGAAGCTGTCGGTGG-3′
PLC*ε*-1	Forwards, 5′-GTGCGGCTGACACTCTGTAA-3′
	Reverse, 5′-CCAGCGTCCTTGAGTTGCTA-3′
GAPDH	Forwards, 5′-AATGGGCAGCCGTTAGGAAA-3′
	Reverse, 5′-GCGCCCAATACGACCAAATC-3′

## Data Availability

The methods and data generated and analyzed in this study are available from the corresponding author (Rui Liu) on reasonable request.
